# A Granular Interaction Framework for Surgical Robotic Digital Twins: Enabling Realistic Segment-Level Manipulation in Extended Reality

**DOI:** 10.1007/s11548-026-03677-1

**Published:** 2026-05-19

**Authors:** An-Nhien Vo, Dhanakumaresh Subramani, Joachim Schmidt, Jan Arensmeyer, Philipp Feodorovici

**Affiliations:** 1 Department of Thoracic Surgery, University Hospital Bonn, Bonn, Nordrhein-Westphalia Germany; 2 Bonn Surgical Technology Center (BOSTER), University Hospital Bonn, Bonn, Nordrhein-Westphalia Germany; 3 Department of Thoracic Surgery, Helios Hospital Bonn/Rhein-Sieg, Bonn, Nordrhein-Westphalia Germany

**Keywords:** Interaction system, Extended reality, Geometric representation, Surgical robotic system, Digital operating room, Digital twin

## Abstract

****Introduction**:**

While Robot-Assisted Surgery (RAS) offers significant clinical advantages, adoption is often hindered by steep learning curves. Extended Reality (XR) and Digital Twins (DT) provide promising solutions for surgical training. However, existing integration pipelines require high-fidelity system-level simulations that address complex kinematic constraints. This paper proposes a modernized framework for integrating RAS into Extended Reality (XR) by transitioning from CAD models to scan-derived digital twins (DTs), as well as a force-based interaction paradigm for realistic simulation.

****Methods**:**

Performance profiling was conducted on the Apple Vision Pro (AVP) to establish empirical hardware benchmarks for polygon counts. Utilizing 3D scanning and manual retopology, we developed a digital surrogate of the Dexter Surgical Robotic System, prioritizing model fidelity within stand-alone XR constraints. Within Unity, we evaluated Rigidbody versus ArticulationBody physics for kinematic stability. This necessitated the development of a force-based interaction system supporting both linear feedback (PD) and geometric constraint-based controllers.

****Results**:**

The scan-derived model achieved a spatial accuracy of 1.6mm relative to the physical system. ArticulationBody physics demonstrated superior numerical stability compared to traditional Rigidbody components. While the linear feedback controller showed higher quantitative precision, it required extensive manual gain tuning; conversely, the geometric constraint-based controller provided high-speed stability without iterative calibration.

****Conclusion**:**

This framework modernizes RAS integration by providing a non-CAD-dependent workflow, a prescriptive testing protocol for mesh decimation, and a force-based interaction system that enhances kinematic realism for clinical training.

## Introduction

Motivated by gains in precision and ergonomics that enhance safety for surgeons and patients [1, 2], the growth of robotic-assisted surgery (RAS) beyond the da Vinci Surgical System (Intuitive Surgical Inc. Sunnyvale, CA, USA) has offered hospitals a broader spectrum of choices [3–5] and spurred a recent surge in academic interest [6] in this area. Yet, these newer systems frequently introduce a learning curve that prolong surgery times [7]. Such inefficiency stems from both operational unfamiliarity [8] and suboptimal planning of the operating room (OR) layout, which often result in intraoperative repositioning as well as restriction on the mobility of the surgical team [9]. In addition, determining incision sites that prevent collisions and avoid joint limits is still largely experience-based, and the distinct mechanical constraints of emerging RAS systems make this task increasingly challenging [10]. Furthermore, patient-specific anatomy requires tailored robotic configurations, further complicating preoperative planning. This variability necessitates more sophisticated planning tools to ensure adequate workspace and reliable instrument access [10].

To address this, digital twins (DT) and extended reality (XR) have emerged as key technologies for enhancing training and preoperative planning [11, 12]. Having a virtual replica of the robotic system reduces reliance on the physical system for planning the OR layout and the surgical team workflow [13], minimizing intraoperative layout modifications that contribute to reduced procedure duration, operational costs, and associated risks [14]. Although work has already begun on the integration of these two technologies [15, 16], their true potential in surgery lies in the development of a digital operating room (DOR). By sharing a common geometric representation and physics system, DT can function as the back-end, while XR serves as the front-end interface.

Geometric representations, which are categorized into grid, point, latent-space, and functional models [17], form the architectural foundation of the DT and XR platforms. In the context of RAS, mesh and computer-aided design (CAD) are typically created using various 3D design software, such as 3DMAX and AutoCAD [18]. These models enable diverse applications, including haptic feedback synthesis [19] and workspace optimization for trocar placement [20]. XR technologies (virtual, augmented, and mixed reality) rely on digital surrogates in the form of meshes for preoperative planning and skill acquisition [21, 22]. When integrated with DT-driven predictive validation, these models also reduce telesurgery latency [23, 24], bridging the gap between immersive training and real-time robotic intervention [11].

To simulate and visualize digital models, DT usually uses simulation programs with built-in physics engines, such as Nvidia Omniverse [25] and game engines such as Unity [19, 26]. Similarly, XR also utilizes a game engine to simulate and visualize the virtual world [11, 15].

Even though integrating DT and XR to boost RAS adoption is feasible, current research often relies on proprietary CAD models that are inaccessible to many clinical teams and require cumbersome conversion pipelines into mesh for XR development platforms like Unity. Furthermore, many XR interaction systems bypass physics engines in favor of rigid kinematic scripts, leading to unrealistic interactions that lack weight and resistance. While the Immersive Robot Interaction System (IRIS) framework [15] streamlines integration, it lacks the joint-level control essential for surgeons to understand movement limits and plan for collision avoidance. Moreover, reliance on specialized controllers in systems like VRDT-RMIS [11] limits compatibility with commercial XR hardware.

Consequently, a gap in the interaction method for robotic simulation for RAS persists: the lack of a hardware-agnostic method to manually position robotic segments in a given OR setup, mirroring the physical positioning of the robot in a matching virtual environment. Thus, we propose a force-driven segment manipulation framework using ArticulationBody physics, allowing intuitive "push-pull" interaction with individual robot segments that closely mimics real OR behavior. This granular control supports precise preoperative tasks like manual collision avoidance and configuration tuning, and helps nurses and surgeons gain kinematic familiarity with new robotic systems, thereby potentially shorten preoperative docking and setup time. Furthermore, we remove the practical hurdle of accessing proprietary data by offering a CAD-independent pipeline, utilizing 3D scanning and manual retopology to create high-fidelity digital surrogates that maintain clinically acceptable spatial accuracy without the need for proprietary data.

We validate our hardware-agnostic framework by developing a virtual OR for the Dexter Surgical Robotic System (Distalmotion SA, Lausanne, Switzerland) on the Apple Vision Pro (AVP). Unlike single-cart systems like the da Vinci or simple cobots [15, 26, 27], Dexter’s modular, multi-base design introduces complex collision risks and patient-specific configuration challenges. Our framework mitigates these hurdles by allowing the surgeon to virtually validate patient-specific configurations before surgery. The successful deployment of the framework demonstrates strong generalization for kinematically complex, distributed robotic platforms. The paper’s specific contributions are:

- A modernized pipeline to generate performance-optimized mesh representations of surgical systems for XR, independent of proprietary CAD data.
- A force-based interaction model that translates physical manipulation paradigms into the virtual environment, enabling granular control of the robotic arm.
- A quantitative validation of physics engine stability (comparing traditional Rigidbody vs. Articulation Body solvers), establishing a standard for kinematic accuracy in DTs for a surgical robotic system.

## Methodology and Implementation

### Acquisition and Optimization of Geometric Representation of the Digital Operating Room

High-precision structured light scanning with the EinScan H2 (Shining 3D, Hangzhou, China) was used for the Dexter Surgical Robotic System, in particular the two independent robotic arms responsible for operating the surgical tools, and the endoscopic camera arm. The other components from the Dexter system (operating console and display stand) and other standard components inside an operating room (the operating table, anesthesia machine, endoscopic tower) were modeled using photogrammetry with MagiScan 3D (MagiScan inc., Wilmington, Delaware, USA). To ensure real-time performance in the Apple Vision Pro (AVP), meshes were processed in Blender (Blender Foundation, Amsterdam, The Netherlands) using the "Decimate" modifier. The complex robotic and endoscopic arms were manually reconstructed as low-polygon models and rigged with a hierarchical segment structure to facilitate physics-based animation within Unity.

To determine the optimal balance between mesh quality and polygon reduction, a stress test was conducted on the AVP to quantify its real-time rendering and physics processing limits. Using Unity, a confined virtual environment was established, where textured "spiky balls" with active physics were instantiated at one-second intervals and allowed to bounce around within the confined space. The frame rate of the system was continuously monitored as the ball count increased, providing a performance baseline to maintain a stable XR experience until the application reached its computational threshold.

**Fig. 1:**
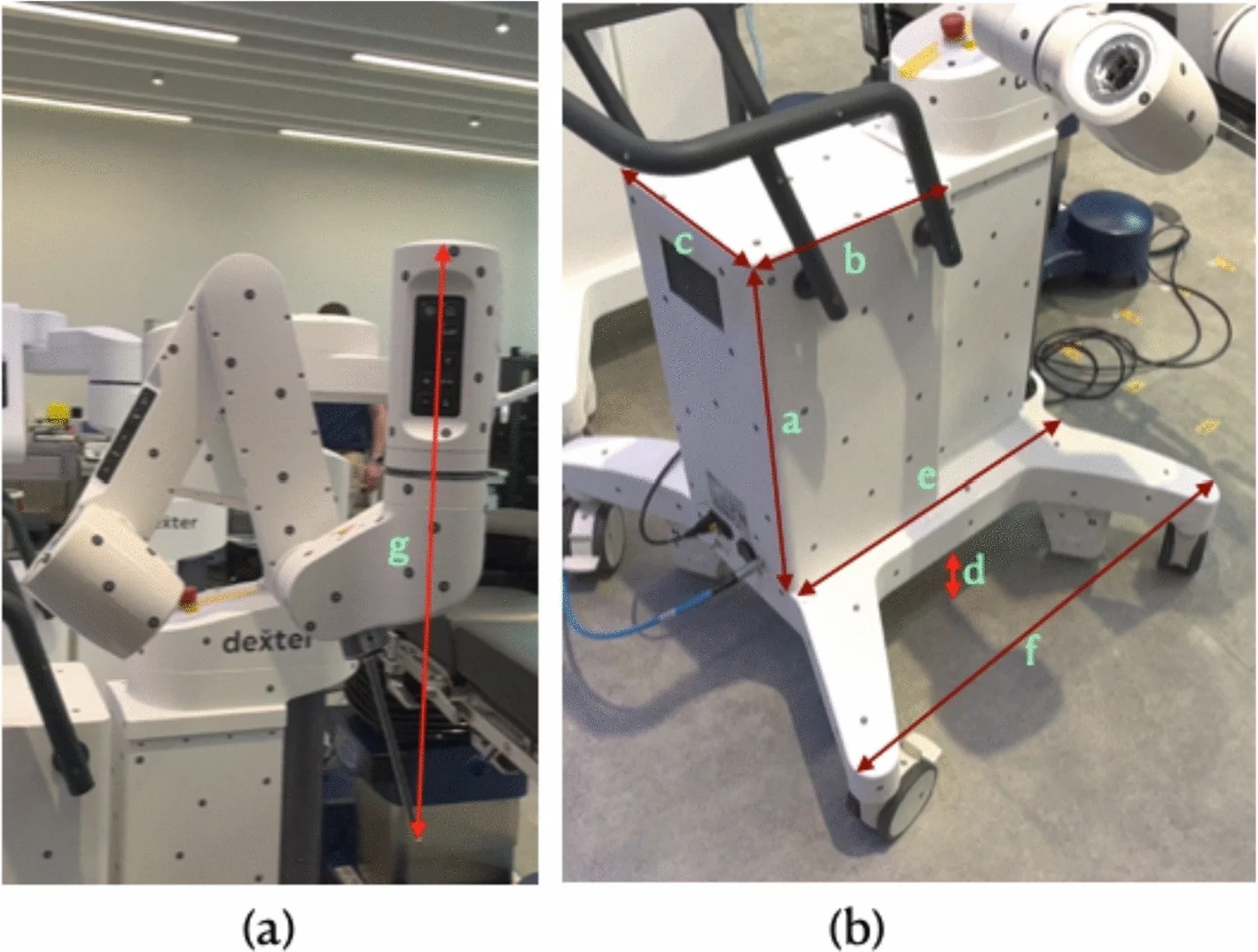
Measurements of the physical Dexter robot to validate against the virtual model

Furthermore, the similarity between the created digital mesh and its physical counterpart needs to be validated to ensure that the virtual object stays true to the physical dimensions within an acceptable tolerance. This requires measuring and comparing the key dimensions of the physical and digital Dexter robots. The specific dimensions to be measured are shown in Fig. 1.

### Choice of simulation system in Unity

While Unity is chosen as the main simulation engine for its efficient development workflow on commercial XR headsets such as the AVP, its default Rigidbody and joint physics are known to be unstable with unrealistic motion. This stems from the use of iterative solvers that treat each segment as an independent rigid body in world space, with joints acting only as constraints to link them, which leads to jittery and bouncy segment motion [28]. Fortunately, Unity introduced ArticulationBody (Articulation) [29] geared for robotics development in 2020, which replaces the traditional RigidBody+Joint combination, offering greater accuracy and stability for robotic kinematics.

To validate stability, virtual copies of the Dexter arm were created using Rigidbody+Joint and Articulation configurations. The model used 1kg segments and nine joints, including an "invisible" trocar joint for closed-chain kinematics. The study focused on the first four manually manipulated segments.

As a control, the joints between Segment 1, 2, 3 and Hinge were rotated at 2$^{\circ }/s$. Stability was tested by applying a downward force to the fourth segment after five seconds. Performance was quantified using three metrics:

- Maximum Deviation: Maximum Euclidean distance of each segment from the control path.
- Y-Axis Deviation: Vertical displacement (which should ideally be zero).
- Settling Time: The time required for the y-axis deviation to return within 5% of its peak.

### Interaction Algorithm for Articulation in Unity

Although ArticulationBody provides high robotic accuracy, it is incompatible with standard XR frameworks that rely on Rigidbody for physics and haptics. We resolved this using a force-based interaction method with impulse-based control, to mimic real world interaction with the robot. This system calculates the acceleration needed to align objects with an interactor, utilizing local coordinate spaces to maintain stability regardless of global movement.

The interaction begins when the interactor first comes into contact with the object. A reference point $P_{local\_first}$ was recorded by converting the position of the interactor at the point of contact into the local space of the object. During each physics update, the current interactor position $P_{world\_interactor}$ is transformed into the local space:

$$\begin{aligned} P_{local\_current} = T_{world \rightarrow local} \cdot P_{world\_interactor} \end{aligned}$$

The local displacement vector, which represents the positional error $\vec {e}_{local}$, is defined as:

$$\begin{aligned} \vec {e}_{local} = P_{local\_current} - P_{local\_first} \end{aligned}$$

Since a force vector F is needed to move the object, where $F = \vec {a}_{global} \cdot m$, $\vec {a}$ is the acceleration required and m is the mass of the object, two types of controller are identified to be viable for our use case: geometric constraint-based controller and linear feedback controller. The job of the controller is to determine the corrective acceleration vector $\vec {a}$ from the current local velocity $\vec {v}_{local}$.

**Fig. 2:**
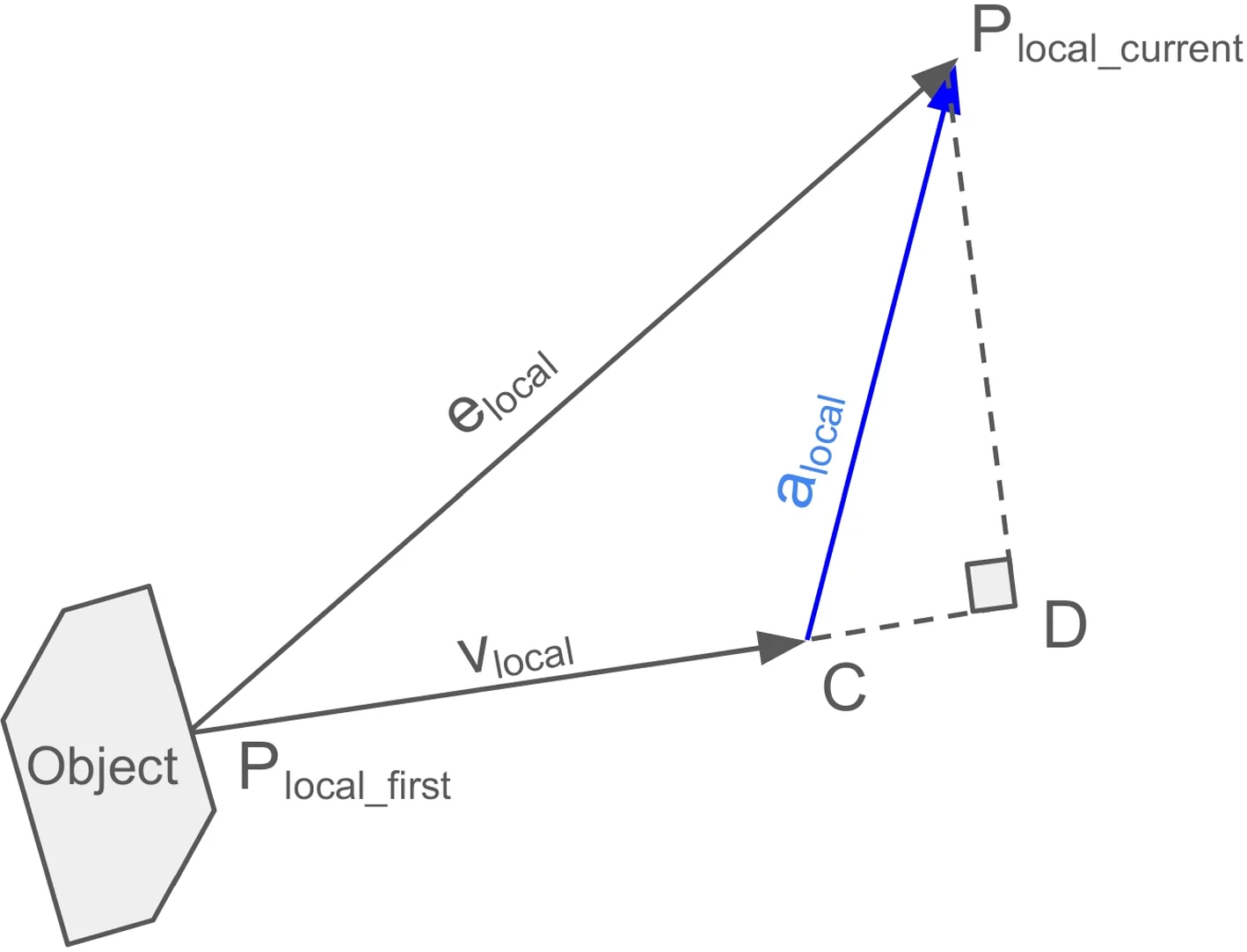
Diagram of how $a_{local}$ is calculated in geometric constrained-based controller

In a geometric constrained-based controller, $\vec {a}_{local}$ is determined using a three-tier logic gate:

1. If $\Vert \vec {v}_{local}\Vert = 0$, the acceleration is mapped directly from the displacement:
  $$\begin{aligned} \vec {a}_{local} = \vec {e}_{local} \end{aligned}$$
2. If the velocity is aligned with the target ($\vec {v}_{local} \times \vec {e}_{local} = 0$), the acceleration would need to consider the current velocity to prevent overshoot:
  $$\begin{aligned} \vec {a}_{local} = \vec {e}_{local} - (P_{local\_first} + \vec {v}_{local}) \end{aligned}$$
3. In general case where velocity is non-zero and non-collinear with $\vec {e}_{local}$, the algorithm uses scalar projection to find the point D on $\vec {v}_{local}$, which is the position on the velocity line closest to $P_{local\_current}$ (Figure 2). The scalar projection t is calculated as:
  $$\begin{aligned} t = \frac{\vec {e}_{local} \cdot \vec {v}_{local}}{\Vert \vec {v}_{local}\Vert ^2} \end{aligned}$$
  The projection point D and the current velocity $\vec {v}_{local}$ endpoint C are defined as:
  $$\begin{aligned} D =P_{local\_first} + t \cdot \vec {v}_{local} \end{aligned}$$
  $$\begin{aligned} C =P_{local\_first} + \vec {v}_{local} \end{aligned}$$
  The final $\vec {a}_{local}$ is derived by summing the velocity correction and the orthogonal attraction:
  $$\begin{aligned} \vec {a}_{local} = (D - C) + (P_{local\_current} - D) \end{aligned}$$

On the other hand, the linear feedback controller determines $\vec {a}_{local}$ by the following equation:

$$\begin{aligned} \vec {a}_{local} = (K_p \cdot \vec {e}_{local}) - (K_d \cdot \vec {v}_{local}) \end{aligned}$$

where the proportional component $K_p$ is the stiffness at 5 and the derivative component $K_d$ is the damping at 1. These values are manually determined through simulation tests.

Finally, $\vec {a}_{local}$ is converted to $\vec {a}_{global}$, before converting to force $F = \vec {a}_{global} \cdot m$ where m is the mass of the object. The force is then applied as a linear force to the center of mass to drive the translation and a positional force at the global coordinate of the point $P_{local\_first}$ to generate the appropriate torque.

To quantify the performance of both controllers, three metrics are used. The duration of energy dissipation is quantified as the time it takes for the moving-average kinetic energy—calculated over a sliding window of 500 milliseconds—to converge and remain below a stability threshold ϵ of 0.005 Joules. Settling time is the time required for the object’s linear speed to reduce and remain below a constant threshold for 1 second. Finally, residual oscillation amplitude measures the largest magnitude of $\vec {e}_{local}$ after the kinetic energy converges.

**Fig. 3:**
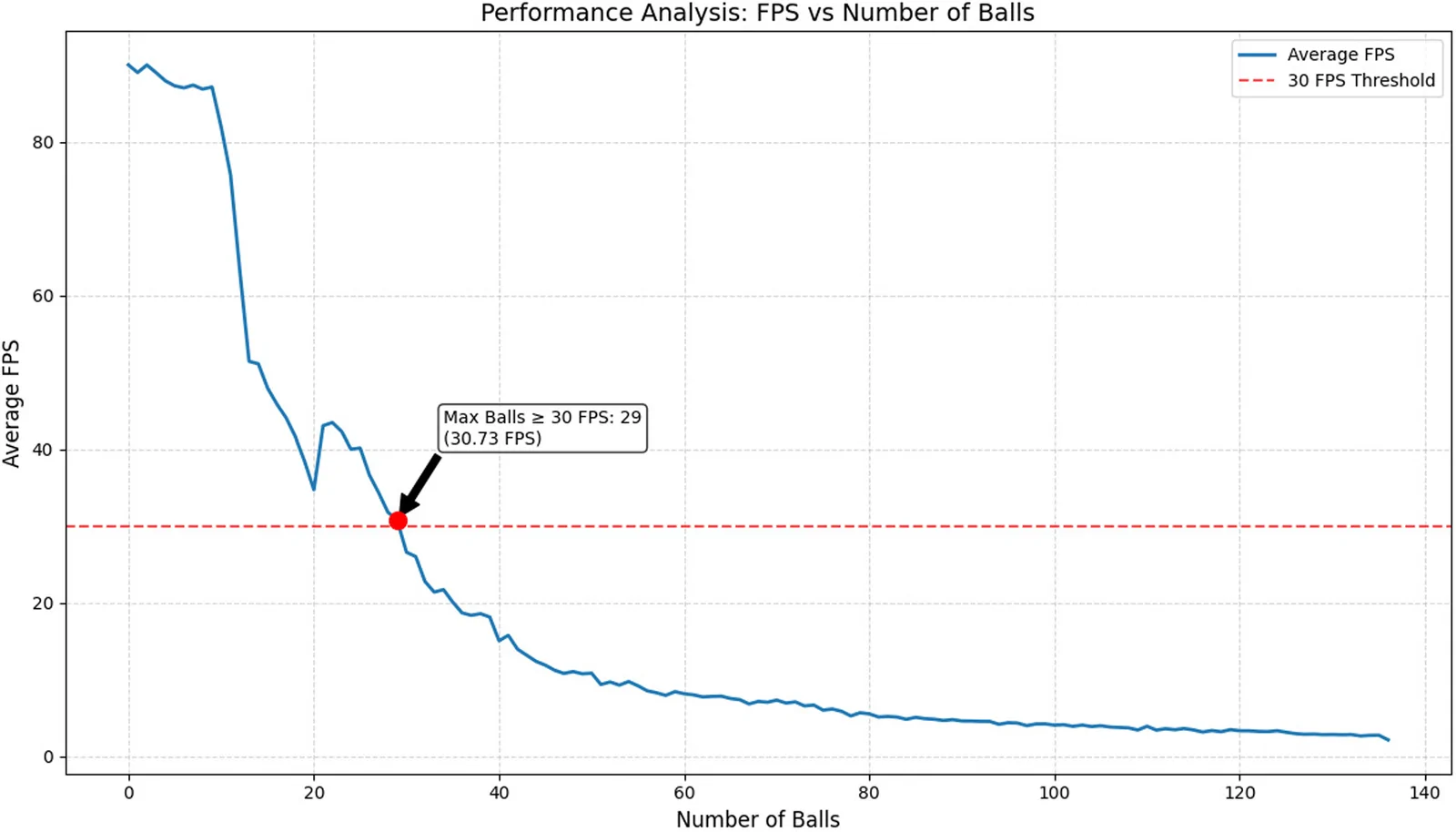
Relationship between the number of bouncing balls and the average frame per second

## Result

### Computation performance of the AVP

Since a frame rate below 30 frames per second will significantly impact the user experience in extended reality, the result from the stress test (Figure 3) showed that the AVP can only handle up to 29 balls before the frame rate drops below 30. As each ball has 215040 triangles, the AVP can handle around 6,236,160 triangles before the user has a unpleasant experience due to low frame rate, and they might be more susceptible to motion sickness.

### Resemblance Accuracy of the Robot Digital Twin

**Table 1:** Difference selected measurement points between Dexter Robot and its physical twin

Measurement Point	a	b	c	d	e	f	g	Average
**Difference between physical robot and digital twin (mm)**	0.9	1.2	0.4	1.7	0.1	3.3	3.7	**1.6**

While the EinScan H2 could produce a highly accurate 3D model, the high polygon count makes it unusable for the AVP, as it will struggle to render the model once the physics simulation is involved. Therefore, the decision was made to sacrifice some accuracy by manually creating a low-polygon model using the scanned model as a reference. From the result in Table 1, an average difference of 1.6mm is negligible and is considered clinically acceptable, for a robot of 90cm by 85cm with a maximum height of 180 cm.

**Table 2:** Comparison of the deviation of the segments from the path when a force is applied

Segment	1		2		3		Hinge	
Physics system	Articulation	Rigidbody	Articulation	Rigidbody	Articulation	Rigidbody	Articulation	Rigidbody
Maximum deviation from path (mm)	0	6.147	0.0224	158.39	0.653	220.687	0.0832	313.765
Maximum deviation along y-axis (mm)	0	5.5	0	22.9	0.01	102.18	0.01	157.81
Settling time along y-axis (s)	0.005	12.688	0.005	13.621	7.652	12.771	0.407	12.747

### Comparison of Physics systems

The result of Table 2 shows that physics for the Articulation is immensely more stable than Rigidbody when an external force perpendicular to the joint movement is applied, as the maximum deviation along the y-axis, which is the same direction as the applied force, is only 0.01mm for Articulation but at 157.81mm for Rigidbody. Another important metric is the time taken for the setup to reduce its deviation on the y-axis to less than 5% of the maximum deviation, called the settling time. The setup using Articulation took a median time of 0.21s to settle, while the setup with Rigidbody took a median time of 12.72s to settle. This shows that Articulation is better suited to robotics applications that require system rigidity Table 3.

**Table 3:** Performance of two different controllers used for interaction system

Controller type	Geometric constraint	Linear feedback
Average energy dissipation rate (s)	4.7980	2.2414
Average settling time (s)	3.1387	2.4407
Average residual oscillation amplitude (mm)	1.201	0.006

### Performance of Interaction Systems

The performance of the interaction algorithm that uses a linear feedback controller generally performed better than the geometric constraint-based controller. For the linear feedback controller, the average energy dissipation rate is at 2.24s, with a settling time of 2.44s and a residual oscillation amplitude of 0.006mm. On the other hand, the geometric constraint-based controller fared slightly worse, as it needs on average 4.80s to dissipate its kinetic energy, its average settling time is at 3.14s, and its average residual oscillation amplitude is at 1.20mm. This shows that both controllers are valid choices to use in the interaction system, and the reason for choosing one over the other would be more nuanced.

## Discussion

XR and DT technologies are pivotal in accelerating the adoption of RAS by facilitating high-fidelity system-level simulations that enhance preoperative spatial planning and clinician familiarity. While the overarching phases of the integration pipeline—geometric acquisition, physics integration, and interaction mapping—remain conceptually unchanged, this paper aims to modernize the practical execution of these steps to align with new technological capabilities.

This paper does not seek to redefine the abstract workflow, but rather to provide a critical methodological update at the implementation level. As surgical robotics transitions toward more complex, often proprietary architectures, the steps of the pipeline require a shift in detail, such as moving from a reliance on a priori CAD models ( which is seldom available) toward high-fidelity 3D scanning and retopology, to using Articulation physics with force-based interaction system. By refining these specific technical layers, we ensure that the pipeline remains viable for modern and diverse surgical robotic systems on modern XR devices such as the AVP. Enhancing these details enables surgeons to move beyond static visualization to direct, immersive interaction with a robot’s digital surrogate, ultimately enabling more precise preoperative planning and reduced intraoperative complications.

**Fig. 4:**
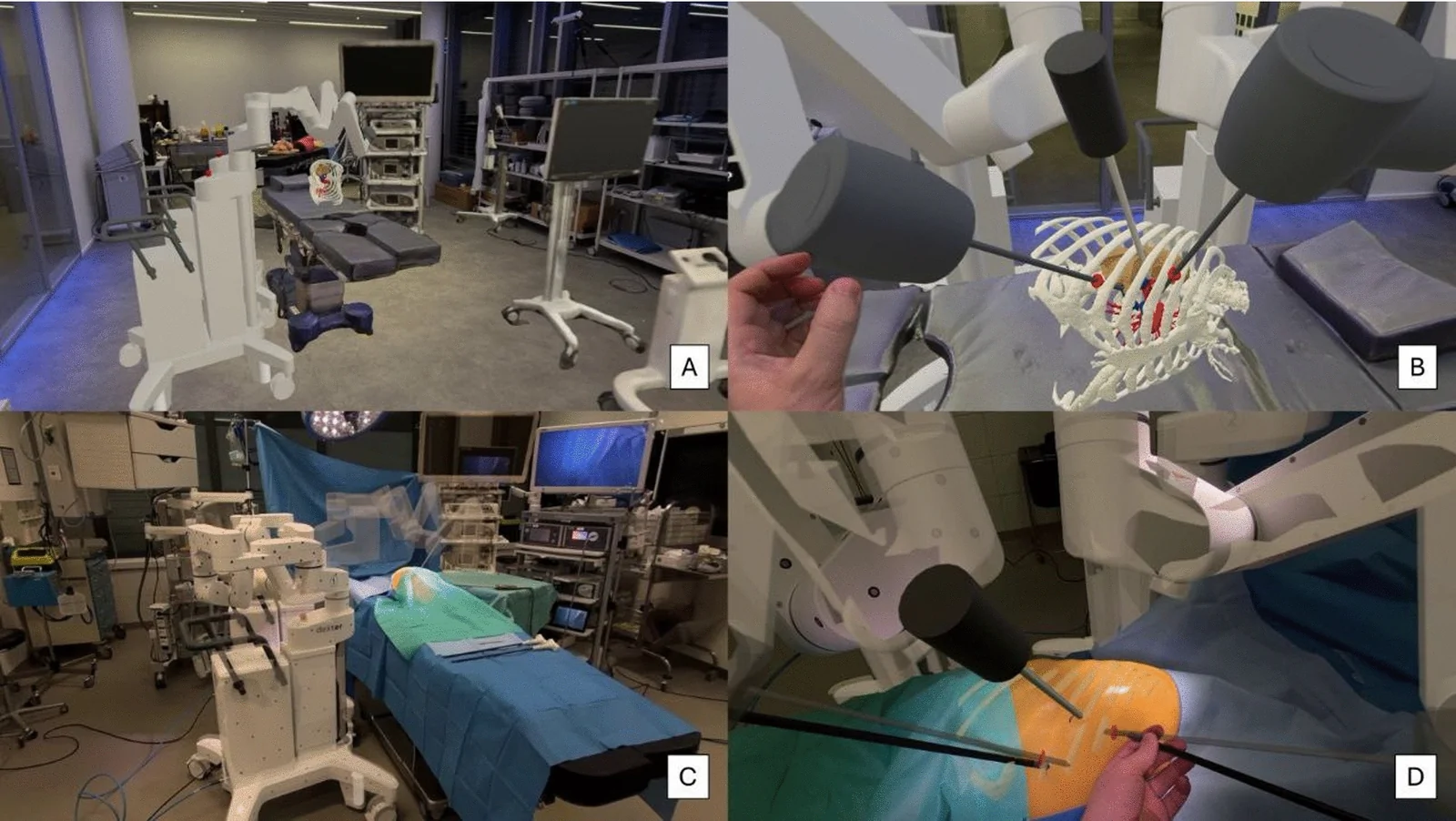
System in used for simulated surgery planning

The virtual OR on the AVP (Figure 4) provides a practical solution to the logistical hurdles of the modular robotic surgical system, where independent arms, such as those of the Dexter system, complicate OR layouts and team mobility. Using a CAD-independent scanning pipeline, the practical hurdle of proprietary data of robotic system from manufacturer that often stalls the adoption of XR in clinical settings can be overcome. Together with the use of force-based interactions, our system allows the surgical team to build intuition and validate docking configurations (4A, 4B), preventing the need for repositioning of high-risk, time-consuming equipment mid-procedure. The ability to project these validated configurations directly into the physical OR (4C, 4D) through mixed reality transforms planning from an abstract task into a shared concrete roadmap for the entire team. Ultimately, this "digital-to-physical" transition directly addresses communication-related installation errors that remain a primary barrier to successful robotic-assisted surgery [30].

The transition from raw 3D scans to interactive digital twins is constrained by XR hardware limits. Our stress test protocol provides a prescriptive mesh optimization framework that pinpoints the polygon-reduction threshold maximizing geometric fidelity within these constraints. Because high-resolution scans are computationally expensive and standard automated decimation fails to preserve key details, we use manual reconstruction for models needing both high fidelity and low weight. This dependence on 3D modeling expertise reduces generalizability, but the scan still offers a geometric template that significantly lowers the cognitive and technical effort needed to create a lightweight, XR-ready asset.

While ArticulationBody offers superior stability for surgical robotics compared to Rigidbody, it lacks native XR interaction support. We addressed this by developing a force-driven interaction layer that translates user input into external forces, enabling intuitive "push-pull" manipulation compatible with both forward and inverse kinematics. This hardware-agnostic framework is compatible with any XR system, as it requires only the position of the interactor.

Since both controllers are viable options, the choice comes down to the developer’s preference for the final product. The linear feedback controller is the preferred choice when the application demands rapid and reactive movement, and the developer has the resources for meticulous gain tuning to achieve optimal results. In contrast, the geometric constraint-based controller offers a robust alternative that eliminates the labor-intensive tuning process. By natively maintaining simulation stability, it serves as an ideal solution for developers seeking a reliable, parameter-free integration.

Ultimately, the decision should be guided by the desired balance between the "finely-tuned" responsiveness of linear feedback controller and the "out-of-the-box" stability of geometric constraints.

### Limitation and Future Works

Although this study establishes a detailed and updated technical foundation, several limitations remain that define a clear roadmap for future research. On a technical level, the current reliance on manual 3D modeling hinders scalability. Future efforts will focus on automating the pipeline to create a fully autonomous system for the generation of digital models.

Currently, the framework lacks empirical quantitative and qualitative evaluations on clinical utility. Future research should prioritize user experience (UX) studies involving surgeons with varying levels of XR and surgical expertise. Specifically, we aim to measure how the force-based interaction model influences the learning curve and the overall duration of preoperative planning. We intend to explore the following avenues:

- **Clinical Efficacy**: Comparative studies measuring planning time and surgeon satisfaction with and without the application created from this framework.
- **Mixed Reality Integration**: Investigating the transition from virtual planning to physical execution by projecting configurations directly into the OR to enhance team communication and also reduce setup time.
- **Training Impact**: Assessing whether force-based systems improve robot familiarity compared to other means of training.

## Conclusion

This paper provides a modernized framework for integrating RAS into XR by transitioning from static CAD models to dynamic, scan-derived DTs. By adopting ArticulationBody architectures and a force-driven interaction model, we achieved the kinematic stability necessary for surgical precision. Our geometric constraint-based controller eliminates the need for manual PD tuning, while our benchmarking methodology ensures high-performance simulation within the limits of mobile XR hardware like the Apple Vision Pro. These advances enable realistic simulation of the DOR for mixed reality, for use in training and preoperative planning for individualized patient with RAS, ultimately improving OR efficiency and the adoption of next-generation robotic technologies.

## Data Availability

Not applicable

## References

[1] Haidegger T, Speidel S, Stoyanov D, Satava RM (2022) Robot-assisted minimally invasive surgery-surgical robotics in the data age. Proc IEEE 110(7):835–846. 10.1109/JPROC.2022.3180350

[2] Picozzi P, Nocco U, Labate C, Gambini I, Puleo G, Silvi F, Pezzillo A, Mantione R, Cimolin V (2024) Advances in robotic surgery: A review of new surgical platforms. Electronics 13(23):4675. 10.3390/electronics13234675

[3] Cepolina F, Razzoli RP (2022) An introductory review of robotically assisted surgical systems. The International Journal of Medical Robotics and Computer Assisted Surgery 18(4):2409. 10.1002/rcs.2409 PMC9540802 35476899

[4] Seetohul J, Shafiee M, Sirlantzis K (2023) Augmented reality (AR) for surgical robotic and autonomous systems: State of the art, challenges, and solutions. Sensors 23(13):6202. 10.3390/s23136202 PMC10347167 37448050

[5] Cannoletta D, Gallioli A, Mazzone E, Gaya Sopena JM, Territo A, Bello FD, Casadevall M, Mas L, Mancon S, Diana P, Palou J, Breda A (2025) A global perspective on the adoption of different robotic platforms in uro-oncological surgery. Eur Urol Focus 11(1):22–25. 10.1016/j.euf.2025.03.016 40158899

[6] Wang H, Zhang J, Xie N, Liu J (2025) Visualized knowledge map of robot application in surgical field: a bibliometric analysis. J Robot Surg 19(1):350. 10.1007/s11701-025-02474-3 40615684

[7] Maruthappu M, Duclos A, Lipsitz SR, Orgill D, Carty MJ (2015) Surgical learning curves and operative efficiency: a cross-specialty observational study. BMJ Open 5(3):006679. 10.1136/bmjopen-2014-006679 PMC4360802 25770229

[8] Power AD, D’Souza DM, Moffatt-Bruce SD, Merritt RE, Kneuertz PJ (2019) Defining the learning curve of robotic thoracic surgery: what does it take? Surg Endosc 33(12):3880–3888. 10.1007/s00464-019-07035-y 31376007

[9] Kennedy S, Fuller P, Cha JS, Carbonell AM, Luo Q, Joseph A (2025) Exploring the impact of the physical environment on robotic-assisted surgery outcomes and processes: A scoping review. Hum Factors 67(10):1011–1045. 10.1177/00187208251333907 40267990

[10] Du Z, Wang W, Wang W, Dong W (2017) Preoperative planning for a multi-arm robot-assisted minimally invasive surgery system. SIMULATION 93(10):853–867. 10.1177/0037549717719336

[11] Cai X, Wang Z, Li S, Pan J, Li C, Tai Y (2023) Implementation of a virtual reality based digital-twin robotic minimally invasive surgery simulator. Bioengineering **10**(11). 10.3390/bioengineering10111302 PMC10669730 38002426

[12] Wah JNK (2025) The rise of robotics and ai-assisted surgery in modern healthcare. J Robot Surg 19(1):311. 10.1007/s11701-025-02485-0 PMC12181090 40540146

[13] Hamad J, Nocera A, Ferrari V (2023) Handling and docking of the Da Vinci surgical robot using mixed reality. In: 2023 IEEE International Conference on Metrology for eXtended Reality, Artificial Intelligence and Neural Engineering (MetroXRAINE), pp. 144–148. 10.1109/MetroXRAINE58569.2023.10405649 . IEEE

[14] Kleinbeck C, Zhang H, Killeen BD, Roth D, Unberath M (2024) Neural digital twins: reconstructing complex medical environments for spatial planning in virtual reality. Int J Comput Assist Radiol Surg 19(7):1301–1312. 10.1007/s11548-024-03143-w PMC11230969 38709423

[15] Jiang X, Yuan Q, Dincer EU, Zhou H, Li G, Li X, Jia X, Schnizer T, Schreiber N, Liao W, Haag J, Li K, Neumann G, Lioutikov R (2025) IRIS: An immersive robot interaction system. In: 9th Annual Conference on Robot Learning. 10.48550/arXiv.2502.03297

[16] Tu X, Autiosalo J, Ala-Laurinaho R, Yang C, Salminen P, Tammi K (2023) TwinXR: Method for using digital twin descriptions in industrial extended reality applications. Frontiers in Virtual Reality 4:1019080. 10.3389/frvir.2023.1019080

[17] Ding H, Seenivasan L, Killeen BD, Cho SM, Unberath M (2024) Digital twins as a unifying framework for surgical data science: the enabling role of geometric scene understanding. Artificial Intelligence Surgery **4**(3). 10.20517/ais.2024.16

[18] Jin L, Zhai X, Xue W, Zhang K, Jiang J, Bodaghi M, Liao W-H (2025) Finite element analysis, machine learning, and digital twins for soft robots: state-of-arts and perspectives. Smart Mater Struct 34(3):033002. 10.1088/1361-665X/adadcd

[19] Hagmann K, Hellings-Kuß A, Klodmann J, Richter R, Stulp F, Leidner D (2021) A digital twin approach for contextual assistance for surgeons during surgical robotics training. Frontiers in Robotics and AI 8:735566. 10.3389/frobt.2021.735566 PMC8491613 34621791

[20] Żelechowski M, Faludi B, Karnam M, Gerig N, Rauter G, Cattin PC (2023) Automatic patient positioning based on robot rotational workspace for extended reality. Int J Comput Assist Radiol Surg 18(11):1951–1959. 10.1007/s11548-023-02967-2 PMC10589133 37296352

[21] Zhang J, Lu V, Khanduja V (2023) The impact of extended reality on surgery: a scoping review. Int Orthop 47(3):611–621. 10.1007/s00264-022-05663-z PMC9841146 36645474

[22] Isikay I, Cekic E, Baylarov B, Tunc O, Hanalioglu S (2024) Narrative review of patient-specific 3D visualization and reality technologies in skull base neurosurgery: enhancements in surgical training, planning, and navigation. Frontiers in Surgery 11:1427844. 10.3389/fsurg.2024.1427844 PMC11287220 39081485

[23] Su Y-P, Chen X-Q, Zhou C, Pearson LH, Pretty CG, Chase JG (2023) Integrating virtual, mixed, and augmented reality into remote robotic applications: A brief review of extended reality-enhanced robotic systems for intuitive telemanipulation and telemanufacturing tasks in hazardous conditions. Appl Sci 13(22):12129. 10.3390/app132212129

[24] Wang J, Barragan JA, Ishida H, Guo J, Ku Y-C, Kazanzides P (2025) A digital twin for telesurgery under intermittent communication. In: 2025 International Symposium on Medical Robotics (ISMR), pp. 218–224. 10.1109/ISMR67322.2025.11025988

[25] Yu Q, Moghani M, Dharmarajan K, Schorp V, Panitch WC-H, Liu J, Hari K, Huang H, Mittal M, Goldberg K, Garg A (2024) Orbit-Surgical: An open-simulation framework for learning surgical augmented dexterity. In: 2024 IEEE International Conference on Robotics and Automation (ICRA), pp. 15509–15516. 10.1109/ICRA57147.2024.10611637

[26] Ho D, Alanazi A, Alqarni S, Lee C, Sutkin G, Lee Y (2025) Embodied surgical intelligence via digital twins: Autonomous trocar insertion. In: International Workshop on Agentic AI for Medicine, pp. 136–145. 10.1007/978-3-032-06004-4_14 . Springer

[27] Trabelsi A, Sandoval J, Mlika A, Lahouar S (2024) Design and implementation of a digital twin for a multi-robot surgical platform. Robotics and Mechatronics: Proceedings of ISRM 2024 158, 207. 10.1007/978-3-031-59888-3_19

[28] Yakovlev A. Use Articulation Bodies to Easily Prototype Industrial Designs with Realistic Motion and Behavior. Unity Technologies. https://unity.com/blog/industry/use-articulation-bodies-to-easily-prototype-industrial-designs-with-realistic-motion-and Accessed 2025-12-20

[29] Featherstone R (2014) Rigid Body Dynamics Algorithms. Springer, New York, NY, USA. 10.1007/978-1-4899-7560-7

[30] BÄcker HC, Freibott CE, Perka C, Weidenbaum M (2020) Surgeons’ learning curve of renaissance robotic surgical system. International Journal of Spine Surgery 14(5), 818. 10.14444/7116 PMC7671434 33097577

